# Biological Context Linking Hypertension and Higher Risk for COVID-19 Severity

**DOI:** 10.3389/fphys.2020.599729

**Published:** 2020-11-19

**Authors:** Caio A. M. Tavares, Matthew A. Bailey, Adriana C. C. Girardi

**Affiliations:** ^1^Geriatric Cardiology Unit, Heart Institute (InCor), University of São Paulo Medical School, São Paulo, Brazil; ^2^Centre for Cardiovascular Science, The Queen’s Medical Research Institute, The University of Edinburgh, Edinburgh, United Kingdom; ^3^Laboratory of Genetics and Molecular Cardiology, Heart Institute (InCor), University of São Paulo Medical School, São Paulo, Brazil

**Keywords:** COVID, hypertension, renin-angiotensin system, hemodynamic factors, inflammation, dipeptidyl peptidase 4

## Abstract

The coronavirus disease 2019 (COVID-19), caused by the severe acute respiratory syndrome coronavirus 2 (SARS-CoV-2), represents a public health crisis of major proportions. Advanced age, male gender, and the presence of comorbidities have emerged as risk factors for severe illness or death from COVID-19 in observation studies. Hypertension is one of the most common comorbidities in patients with COVID-19. Indeed, hypertension has been shown to be associated with increased risk for mortality, acute respiratory distress syndrome, need for intensive care unit admission, and disease progression in COVID-19 patients. However, up to the present time, the precise mechanisms of how hypertension may lead to the more severe manifestations of disease in patients with COVID-19 remains unknown. This review aims to present the biological plausibility linking hypertension and higher risk for COVID-19 severity. Emphasis is given to the role of the renin-angiotensin system and its inhibitors, given the crucial role that this system plays in both viral transmissibility and the pathophysiology of arterial hypertension. We also describe the importance of the immune system, which is dysregulated in hypertension and SARS-CoV-2 infection, and the potential involvement of the multifunctional enzyme dipeptidyl peptidase 4 (DPP4), that, in addition to the angiotensin-converting enzyme 2 (ACE2), may contribute to the SARS-CoV-2 entrance into target cells. The role of hemodynamic changes in hypertension that might aggravate myocardial injury in the setting of COVID-19, including endothelial dysfunction, arterial stiffness, and left ventricle hypertrophy, are also discussed.

## Introduction

The severe acute respiratory coronavirus 2 (SARS-CoV-2) infection, named coronavirus disease 2019 (COVID-19), was initially described as a series of cases of atypical pneumonia arising in Wuhan, China, in December 2019 (Zhu et al., 2020). The rapid spread of COVID-19 in many countries worldwide has given rise to a global public health crisis of unprecedented proportions in the modern era. As of October 9, 2020, the SARS-CoV-2 has infected 36,669,238 individuals, with 1,063,863 deaths globally (Dong et al., 2020).

The clinical spectrum of COVID-19 ranges from asymptomatic infection to mild or moderate respiratory and associated symptoms (cough, sore throat, nasal congestion, myalgia, arthralgia, headache, shortness of breath) (Guan et al., 2020b) to severe pneumonia accompanied by multiorgan failure which may result in death. Accumulated evidence from the first months of the COVID-19 pandemic has also linked several risk factors with the development of severe morbidity and mortality, such as advanced age, male gender, and the coexistence of underlying chronic diseases. Indeed, the presence of comorbidities, especially hypertension, have been consistently reported as more common among patients with COVID-19 in severe conditions, admitted to the intensive care unit (ICI), who received mechanical ventilation or died, than among patients with mild symptoms (Guan et al., 2020b; Wang et al., 2020; Wu et al., 2020; Zhou et al., 2020a).

Hypertension represents one of the most prevalent comorbidities in patients with COVID-19. Since the first observational data available from China, in early March, hypertension has emerged as a potential risk factor for COVID-19 severity and mortality in different cohorts (Guan et al., 2020b; Zhang et al., 2020; Zhou et al., 2020a). With the pandemic progression worldwide, the association of hypertension and unfavorable outcomes was also seen in other countries such as Italy (Grasselli et al., 2020; Mancia et al., 2020) and the United States (Garg et al., 2020). However, at present, the precise impact of hypertension *per se* on COVID-19 severity is yet to be defined. This review aims to present the biological plausibility linking hypertension and higher risk for COVID-19 severity. To this end, we discuss how cellular, molecular, and functional alterations that underlie the pathophysiology of hypertension can impact the severity of the SARS-CoV-2 infection, thereby predisposing hypertensive patients to more complicated clinical outcomes.

## Role of the Renin-Angiotensin System (RAS)

The RAS is a key player both in the SARS-CoV-2 transmissibility and in the pathophysiology of hypertension. It consists of a complex network of precursors, enzymes, effector peptides, and receptors that exerts a vital role in blood pressure control, extracellular volume homeostasis, and cardiac function, among several other physiological processes. Abnormal activation of RAS components, ultimately leading to the upregulation of angiotensin II (Ang II) and activation of its angiotensin II type 1 receptor (AT1R), contribute to the development and progression of hypertension (Carson et al., 2001; Dahlof et al., 2002; Crowley et al., 2005, 2006; Gurley et al., 2011).

Angiotensin-converting enzyme 2 (ACE2), a type I integral membrane protein, is a homolog of angiotensin-converting enzyme (ACE), the central enzyme of classical RAS (Tipnis et al., 2000). ACE2 is expressed in organs that are important for blood pressure control such as kidneys, vessels, brain, and heart, where it hydrolyzes Ang II (Donoghue et al., 2000; Tipnis et al., 2000). ACE2 is also found in the lungs, small intestine, ovaries, and testicles (Tipnis et al., 2000). Additionally, ACE2 has been identified as a functional receptor for the SARS-CoV-2 host cell entry (Hoffmann et al., 2020) as well as for its predecessor SARS-CoV (Kuba et al., 2005). Binding of the viral spike (S) protein of the SARS-CoV-2 to the extracellular domain of ACE2 triggers conformational changes that destabilize the membrane allowing the internalization of the SARS-CoV-2 along with ACE2, leading to ACE2 cell surface expression downregulation, viral replication and cell-to-cell transmission (Heurich et al., 2014; Hoffmann et al., 2020). During this process, the cleavage of the S protein by host cell proteases, including the transmembrane serine protease 2 (TMPRSS2), is essential for viral infectivity (Iwata-Yoshikawa et al., 2019). As such, TMPRSS2 constitutes a potential target for the treatment of SARS-CoV-2 infected patients.

As a bioactive component of the RAS, ACE2 functions as a counterregulatory enzyme, converting Ang II to Ang-(1-7). This heptapeptide binds to the Mas receptor (MasR), modestly reducing blood pressure, promoting vasodilation, increasing excretion of sodium and water by the kidneys, and exerting anti-inflammatory and antioxidant effects (Santos et al., 2018). These actions are directly opposed to those induced by the activation of the ACE/Ang II/AT1R axis. ACE converts Ang I to Ang II, which in turn acts on the AT1R, increasing blood pressure, inducing vasoconstriction, increasing renal tubular salt and water reabsorption, and increasing the production of reactive oxygen species (ROS) that promote inflammation and fibrosis (Benigni et al., 2010). The ACE/Ang II/AT1R and ACE2/Ang-(1-7)/MasR pathways are co-expressed in most tissues and act in an autocrine and paracrine manner. Thus, the balance between these pathways determines, at least in part, whether or not tissue damage will occur in response to pathological stimuli.

The kidney is a target for end-organ damage in hypertension, plays an active role in the pathogenesis of hypertension, and it is one of the sites of the highest levels of expression of ACE2 (Gembardt et al., 2005). Several studies have found that the protein and mRNA abundance, as well as the activity of ACE2, are reduced in the kidneys of experimental models of hypertension, including spontaneously hypertensive rats, renin transgenic hypertensive rats, aldosterone/NaCl-induced hypertension and the model of 2 kidneys 1 clip (2K1C) hypertensive rats (Soler et al., 2013). In mice on the C57BL/6 genetic background, (Gurley et al., 2006) have found that ACE2 deficiency was associated with a significant increase in blood pressure of ∼7 mmHg and that the absence of ACE2 considerably enhanced the severity of Ang II-dependent hypertension. Moreover, ACE2-deficient mice chronically treated with Ang II infusion displayed a more than 5-fold higher renal Ang II concentration than Ang II-treated wild-type animals, thereby suggesting that the more severe hypertension in ACE2-deficient mice may be attributed to an impaired metabolism of Ang II in the kidney (Gurley et al., 2006). Notably, Ang II upregulates ACE and downregulates ACE2 expression in human proximal tubule cells via an AT1R-mediated mechanism (Koka et al., 2008), thereby suggesting that ACE and ACE2 may be regulated in a balanced manner, which can be mediated via the local Ang II concentration. This synergistic regulation is observed in renal biopsies from humans, in which the ACE to ACE2 ratio is significantly higher in subjects with hypertension than in subjects without hypertension (Wakahara et al., 2007). The human kidney is a target for the SARS-CoV-2 infection (Braun et al., 2020; Puelles et al., 2020). Acute kidney injury (AKI) has been observed in COVID-19 patients, and it is considered a marker of COVID-19 severity and an adverse prognostic factor for survival (Cheng et al., 2020). Renal Ang II overactivity in the setting of hypertension and potentiated by SARS-CoV-2 induced ACE2 internalization, may contribute to the pathogenesis of AKI in severely ill patients with COVID-19. Favoring this hypothesis are the findings of a prospective cohort study of 701 patients with COVID-19 conducted by Cheng et al. (2020). These authors investigated the association between inpatient use of medications and the development of AKI among patients with COVID-19. It was observed that none of the patients who were taking RAS inhibitors on admission or during hospitalization for COVID-19 developed AKI (Guan et al., 2020a).

Angiotensin-converting enzyme 2 expression is relatively abundant in the heart, where it can be found in cardiomyocytes endothelial cells, and fibroblasts (Santos et al., 2018). Crackower et al. (2002) have found that ACE2 knockout mice display increased heart content of Ang II and cardiac dysfunction characterized by a decrease in fractional shortening with slight ventricular dilation. Moreover, these authors have observed that cardiac dysfunction of ACE2 knockout mice progressed with age, and it was more pronounced in males than in females. The fact that cardiac phenotype and increased Ang II levels were completely reversible by deleting the ACE gene in ACE2 knockout mice strengths the notion that cardiac function is modulated by the balance between ACE and ACE2, and that the increase in local cardiac Ang II was involved in cardiac impairment (Crackower et al., 2002). The cardiac effects of ACE2 remain under debate since ACE2 deletion mediated-cardiac dysfunction was not observed by Gurley and colleagues. On the other hand, it is well accepted that increased cardiac Ang II, generated by cardiac ACE, drives left ventricular hypertrophy (LVH) in multiple settings, including hypertension (Sadoshima and Izumo, 1993; Crowley et al., 2006; Ainscough et al., 2009). Therefore, patients with hypertension are particularly susceptible to the imbalance between the ACE/Ang II/AT1R, and ACE2/Ang-(1-7)/MasR, further intensified by myocardial SARS-CoV-2-mediated ACE2 internalization (Huentelman et al., 2005). Indeed, loss of surface ACE2 in cardiac cells may be one of the underlying causes of acute, and perhaps long-term, exacerbation of cardiovascular disease in hypertensive patients infected with SARS-CoV-2.

Extrapolating data from SARS-CoV to SARS-CoV-2, one may postulate that the imbalance in the signaling and actions of products of ACE/ACE2, generated by the loss of ACE2 cell surface expression due to SARS-CoV-2 infection, may lead to severe acute respiratory failure in COVID-19 (Kuba et al., 2005). The existence of a causal relationship between the imbalanced ACE/ACE2 axis and the acute respiratory distress syndrome has been established through the use of genetically modified animals (Imai et al., 2005; Kuba et al., 2005). Imai et al. (2005) have found that acute lung injury induced by acid aspiration results in decreased expression of ACE2 and increased lung content of Ang II in wild-type mice. Additionally, ACE2 knockout mice with severe acute lung injury induced by acid aspiration or sepsis displayed a higher rate of mortality and lung failure than wild type mice with severe acute lung injury (Imai et al., 2005). Conversely, the genetic deletion of ACE in ACE2 knockout mice significantly attenuated these outcomes, demonstrating that ACE/Ang II drive severe lung, whereas ACE2 protects against it. The levels of ACE2 gene expression appear to be upregulated in the lung of patients with pulmonary hypertension when compared to controls (Pinto et al., 2020), however, to our knowledge, the modulation of lung ACE to ACE2 ratio in essential arterial hypertension remains elusive.

## Role of RAS Inhibitors

During the early beginning of the COVID-19 pandemic, concerns emerged that RAS inhibitors, cornerstone treatment of several cardiovascular diseases, including hypertension, could promote viral interaction with host cells, leading to increased cell entry, viral replication and thereby COVID-19 exacerbation (Diaz, 2020; Esler and Esler, 2020). These concerns were primarily based on findings that ACE inhibitors (ACEi) or angiotensin II type 1 receptor (ARB) upregulate the expression and activity of ACE2, the SARS-CoV-2 receptor, in the kidneys and heart of experimental models of hypertension (Ferrario et al., 2005; Jessup et al., 2006; Wang et al., 2016).

As the pandemic evolved, several observational studies indicated that ACEi/ARBs use are not a risk factor for disease severity and may actually be related to milder disease and better outcomes (Supplementary Table S1) possibly by attenuating the imbalance between ACE/Ang II/AT1R and ACE2/Ang-(1-7)/MasR, reducing pathogenic inflammation and multiorgan injury. Also, evidence from population studies suggests that RAS inhibitors neither increase the risk of SARS-CoV-2 infection in patients with hypertension nor negatively impact the disease severity in those who are infected, establishing its safety and reinforcing that they should not be switched/stopped during the pandemic (Mancia et al., 2020; Mehta et al., 2020; Reynolds et al., 2020).

Ongoing clinical trials will add crucial information on the impact of RAS on COVID-19 severity. Currently, several studies are registered in the clinicaltrials.gov platform aiming to investigate the effects of ACEi/ARBs replacement or withdrawn on patients with COVID-19 (Table 1), the impact of ACEi/ARBs initiation in patients without hypertension on the risk of COVID-19 infection and severity (Table 2) and whether modulation of RAS by other agents with antihypertensive actions [AT1R biased agonist, Ang-(1-7) analogs, DPP4 inhibitors or recombinant ACE2] can impact COVID-19 outcomes (Table 3).

**TABLE 1:** Ongoing randomized trials comparing ACEi/ARBs replacement or withdrawal in patients with COVID-19.

Category	NCT number	Study design	Acronym	Intervention arm	Study population	Target enrollment	Primary outcome measure
ACEi ARB replacement or withdraw	NCT04330300	Randomized, open-label	CORONACION	Switch RAAS inhibitor to alternative medication	Ambulatory hypertensive patients without COVID-19	2414	Composite: death, mechanical ventilation, ICU hospitalization or hospitalization for NIV
	NCT04351581	Randomized, single-blind (outcomes assessor)	RASCOVID-19	Discontinue RAAS inhibitor and start other medication as needed	Hospitalized patients with COVID-19 and use of RAAS inhibitors	215	Composite: death and days out of hospital within 14 days of recruitment
	NCT04353596	Randomized, single-blind (outcomes assessor)	ACEI-COVID	Stoping or replacing ACEi/ARB	COVID-19 infection ≤5 days	208	(1) SOFA/Death (2) ICU/MV/Death
	NCT04364893	Randomized, open-label	BRACE-CORONA	Temporally discontinuation of ACEi/ARB for 30 days	Hospitalized patients with COVID-19	700	Composite: days alive and out of hospital at 30 days
	NCT04329195	Randomized, open-label	ACORES-2	Discontinuation of RAS blocker	Hospitalized patients with COVID-19	554	Time to clinical improvement on a seven-category ordinal scale
	NCT04338009	Randomized, single-blind (participant)	REPLACECOVID	Discontinuation of ACEi/ARB	Hospitalized patients with COVID-19	152	Hierarchial/Composite: (1) time to death; (2) days at ECMO/MV;(3) days supported by RRT/VAD; (4) modified SOFA

*BP, blood pressure ECMO, extracorporeal membrane oxygenation; ICU, intensive care unit; NIV, non-invasive ventilation; MV, mechanical ventilation; RRT, renal replacement therapy SOFA, Sequential Organ Failure Assessment; VAD, vasoactive drugs. For up-to-date information search the clinicaltrials.gov platform.*

**TABLE 2:** Ongoing randomized trials comparing ACEi/ARBs initiation to mitigate COVID-19 severity in patients with COVID-19.

Category	NCT number	Study design	Acronym	Intervention arm	Study population	Target enrollment	Primary outcome measure
ACEi or ARB initiation ACEi/ARB initiation	NCT04345406	Randomized, open-label	N/A	ACE inhibitors	Patients with COVID-19 without contra-indication to ACE inhibitors	60	Number of patients with virological cure
	NCT04366050	Randomized, double-blind, placebo-controlled	RAMIC	Ramipril 2.5 mg for 14 days	Hospitalized patients or in a emergency department with COVID-19	560	Composite: death, need for ICU admission or MV
	NCT04355429	Randomized, open-label	CAPTOCOVID	Captopril 25 mg by nebulization	Hospitalized patients with COVID-19 needing oxygen	230	Ventilator free survival at 14 days
	NCT04360551	Randomized, double-blind, placebo controlled	N/A	Telmisartan 40 mg	Outpatients with COVID-19	40	Maximal clinical severity on a seven-category ordinal scale
	NCT04335786	Randomized, double-blind, placebo-controlled	PRAETORIAN-COVID	Valsartan 80 to 160 mg titrated by blood pressure	Hospitalized patients with COVID-19	651	Composite: death, mechanical ventilation or ICU admission
	NCT04394117	Randomized, single-blind (outcomes assessor)	CLARITY	Initiation of an ARB or switching from non-RAAS inhibitor to ARB	Confirmed COVID-9	605	Improvement on a seven-category ordinal scale
	NCT04340557	Randomized, open-label	N/A	Losartan 12.5 mg up titrated according to BP	Hospitalized patients with COVID-19 and mild to moderate hypoxia	200	Need for MV
	NCT04312009	Randomized, double-blind, placebo-controlled	N/A	Losartan 50 mg daily	Hospitalized patients with COVID-19 requiring oxygen therapy	200	The difference in P/F ratio at 7 days
	NCT04343001	Randomized, factorial design (2 × 2 × 2), open-label	CRASH-19	Losartan 100 mg daily Other interventions: Aspirin, Simvastatin	Hospitalized patients with COVID-19	10000	Mortality up to 28 days
	NCT04328012	Randomized, double-blind, placebo-controlled, 4 groups (parallel)	COVIDMED	Losartan 25 mg daily Other interventions: Lopinavir/Ritonavir, Hydroxychloroquine	Hospitalized patients with COVID-19	4000	The difference in the ordinal scale of disease severity
	NCT04359953	Randomized, open-label, 4 groups (parallel)	COVID-Aging	Telmisartan 40 mg twice daily Other interventions: Azithromycin, Hydroxychloroquine	Hospitalized patients with COVID-19 and age ≥75 years or ≥60 years if dementia	1600	Mortality up to 14 days
	NCT04351724	Randomized, open-label, adaptative trial	ACOVACT	RAS Blockade substudy Candesartan 4 mg daily, uptitrated Other interventions: Chloroquine, Lopinavir/Ritonavir, Rivaroxaban and Clazakizumab	Hospitalized patients with COVID-19 and blood pressure ≥120/80 mmHg	500	Sustained clinical improvement on a seven-category ordinal scale
	NCT04356495	Randomized, open-label, multi-arm multi-stage trial	COVERAGE	Telmisartan 20 mg daily Other interventions: Hydroxychloroquine, Imatinib, and Faviparavir	Outpatients with COVID-19	1057	Primary outcomes: 1- Mortality up to 14 days 2- Need for hospitalization up to 14 days
	NCT04447235	Randomized, double-blind, placebo-controlled	TITAN	Ivermectin plus Losartan 50 mg daily	Cancer patients with COVID-19	176	Composite: mortality, need for MV or ICU admission up to 28 days
	NCT04311177	Randomized, double-blind, placebo controlled	N/A	Losartan 25 mg	Symptomatic COVID-19 infection	516	Hospital admission up to 15 days
	NCT04355936	Randomized, open-label	N/A	Telmisartan 80 mg twice daily	COVID-19 infection	400	CRP at days 1.8 and 15
	NCT04428268	Randomized, double-blind	N/A	Losartan 25 mg twice daily Chloroquine vs. Chloroquine/Losartan	Hospitalized patients with COVID-19	20	Mortality up to 28 days

**TABLE 3:** Ongoing clinical trials testing the hypothesis that modulation of RAS components can impact COVID-19 severity.

Category	NCT number	Study design	Acronym	Intervention arm	Study population	Target enrollment	Primary outcome measure
Recombinant ACE2	NCT04382950	Randomized, open-label	N/A	Recombinant ACE2 infusion plus aerosolized isotretinoin	Hospitalized patients with COVID-19 and respiratory failure	24	Fever
	NCT04375046	Randomized, open-label	Bacterial ACE2	Recombinant ACE2 infusion	Hospitalized patients with COVID-19	24	(1) Fever (2) Viral load
	NCT04335136	Randomized, double-blind, placebo-controlled	APN01-COVID-19	Recombinant ACE2 infusion	Hospitalized patients with COVID-19	200	Composite: death or mechanical ventilation up to 28 days or hospital discharge
Biased agonist of AT1R	NCT04419610	Randomized, double-blind, placebo-controlled	N/A	TRV027 at 12 mg/hour until discharge or 7 days	Hospitalized patients with COVID-19	60	Mean change from baseline D-dimer at day 8
Ang 1-7 analogs	NCT04332666	Randomized, double-blind, placebo controlled	ATCO	Angiotensin-(1-7) infusion (venous) of 0.2 mcg/Kg/h for 48h	Hospitalized patients with COVID-19 respiratory failure and MV	60	Composite: mortality and MV-free days
	NCT04375124	Non-randomized, open label	N/A	angiotensin peptide (1-7) derived plasma	Hospitalized patients with COVID-19	20	Mortality up to 4 months
	NCT04401423	Randomized, double-blind, placebo-controlled	TXA COVID-19 Clinical Trial	TXA127 0.5 mg/kg per day	Hospitalized patients with COVID-19 requiring oxygen therapy	100	1-Acute kidney injury up to 7 days 2-Need for VM up to 7 days
DPP4 inhibitors	NCT04341935	Randomized, open-label	N/A	Linagliptin 5 mg daily	Hospitalized patients with COVID-19	20	Changes in glucose levels
	NCT04371978	Randomized, open-label	N/A	Linagliptin 5 mg daily	Hospitalized patients with COVID-19	100	Time to clinical improvement (WHO scale of COVID-19)

*Ang-1-7, Angiotensin-1-7; AT1R, Angiotensin II type 1 Receptor; DPP4, Dipeptidyl peptidase 4.*

## Inflammation

There is strong evidence from human and experimental studies to show that chronic hypertension accrues sustained, low-grade inflammation, stimulating the adaptive immune system. This may reflect tissue damage as a consequence of sustained high blood pressure, but experimental evidence also points to the role of the immune system in the generation of hypertension. Indeed, cells of the immune system, which contribute importantly to normal blood pressure homeostasis, may operate pathogenically in hypertension, contributing to pressure-dependent and independent organ damage (Mattson, 2019). Despite intense research, a unifying, mechanistic understanding of the interaction in health and disease has yet to emerge. The innate immune system has some “protective” roles: macrophages, for example, regulate extracellular fluid volume by buffering the release of salt from the skin for renal excretion (Machnik et al., 2009). Monocytes/macrophages can also scavenge reactive oxygen species (Rosenblat et al., 2013) and have a role in clearing vasoactive peptides such as endothelin-1 (Czopek et al., 2019), influencing local vasomotor tone and blood pressure. Depletion of monocytes/macrophages, or impairing their ability to clear endothelin-1, increases blood pressure over a few days in humans and mice (Guzik et al., 2007; Abais-Battad et al., 2018), particularly in the setting of a pre-existing challenge to blood pressure such as high salt or Ang II infusion. Conversely, T-cells and B-cells depletion are protective, reducing hypertension and vascular free-radical production in experimental models (Guzik et al., 2007; Abais-Battad et al., 2018). Thus, these cells of the adaptive immune system appear to be “pro-hypertensive” and experimentally, re-population of the T-cell pool restores the full hypertensive response to chronic Ang II infusion (Fehrenbach et al., 2020). Mechanistically, high blood pressure promotes T-cells activation, increasing their ability to invade organs such as the kidney that are susceptible to barotrauma (Itani et al., 2016). This invasive aspect appears to be directly related to pressure, rather than hormonal aspects such as RAS activation that may contribute to hypertension: preventing the pressure rise significantly reduces T-cell and B-cell infiltration (Shimada et al., 2020).

However, the picture is undoubtedly much more complex. For example, in the long-term, reducing the ability of macrophages to clear vasoactive endothelin-1 does not aggravate hypertensive injury, but unexpectedly protects against end-organ damage. This, in part, reflects the repolarizing of cells to an anti-inflammatory phenotype (Guyonnet et al., 2020). The role of the adaptive immune system is similarly, nuanced, and non-genomic modifiers may influence the “pro-hypertensive” phenotype of the T-cell (Seniuk et al., 2020).

Given the prevalence of hypertension in the general population, it is not surprising that this is a common comorbidity in patients hospitalized with coronavirus (Richardson et al., 2020). However, pre-existing hypertension increases the risk of developing severe disease and also of death (Zuin et al., 2020). How hypertension causes poor clinical outcomes in COVID-19 is not understood, but the intersection of blood pressure homeostasis and the immune system may be important. Certainly, SARS-CoV-2 infection features systemic inflammation and accumulation of inflammatory cytokines, the extent of which is strongly implicated in patient outcome (Huang et al., 2020). Viral interaction with ACE2 provides a further pivot point for local inflammation since ACE2 converts pro-inflammatory Ang II to Ang-(1-7), which has anti-inflammatory roles. It is evident that the inflammation response to COVID-19 is amplified in hypertensives compared to normotensive controls (Yang et al., 2020). In humans, high levels of systemic inflammation driven by infection induce a short-lived, extensive endothelial dysfunction (Hingorani et al., 2000) that would be anticipated to transiently increase cardiovascular risk. It is not difficult to imagine that this risk would be exaggerated for individuals with a vulnerable cardiovascular system, such as those with hypertension. Of importance, in patients receiving antihypertensive medicines, those on ACEi or ARBs had reduced levels of inflammatory biomarkers (C-reactive protein and procalcitonin) and better outcomes than those on other antihypertensive medication (Yang et al., 2020). These outcomes from a retrospective, single-center cohort (Wuhan, China) study give some insight suggesting that immune system/blood pressure interactions are important for COVID-19 severity, and also that ACEi, may have beneficial cardiovascular effects in this setting beyond blood pressure control. Indeed, targeting excessive inflammation is an attractive strategy to improve the health of the arterial cardiovascular system (Zanoli et al., 2020) and may be particularly relevant in understanding cardiovascular risk in COVID-19.

## Hemodynamic Factors

### Endothelial Dysfunction

Endothelial cells play a vital role in cardiovascular homeostasis by controlling vasomotor tone, maintaining vascular integrity, exerting barrier protecting effects, and preventing platelet and leukocyte adhesion and aggregation (Deanfield et al., 2007). It also regulates fibrinolysis and the coagulation cascade, provides antiproliferative and anti-inflammatory actions, and protects against oxidative stress (Deanfield et al., 2007). In turn, abnormalities of the vascular endothelium significantly contribute to a plethora of cardiovascular disorders.

A large body of evidence demonstrates the presence of endothelial dysfunction in patients with hypertension (Watson et al., 2008). Endothelial dysfunction is characterized by imbalanced vasodilation and vasoconstriction, elevated ROS and pro-inflammatory mediators, as well as reduced bioavailability of nitric oxide (NO) (Deanfield et al., 2007; Watson et al., 2008). As aforementioned, Ang II, via AT1R, is a potent activator of oxidative and inflammatory cascades, the primary mediators of endothelial dysfunction. Under physiological conditions, however, the ACE2/Ang-(1-7)/MasR axis stimulates the activity of the endothelial NO synthase, increasing NO production. Adding up to this effect, Ang-(1-7) decreases the activity of nicotinamide adenine dinucleotide phosphate (NADPH) oxidase stimulated by Ang II, directly modulating the generation of reactive ROS (Sampaio et al., 2007).

Many severe COVID-19 patients show signs of a cytokine storm that could be aggravated due to overactivation of Ang II, increased production of ROS, and a preexistent pro-inflammatory state, features of hypertension associated-chronic endothelial dysfunction. In fact, endotheliitis and an increase in D-dimer (a marker of activation of coagulation and fibrinolysis) have been described in the pathological findings of patients with COVID-19 (Tang et al., 2020; Varga et al., 2020). Therefore, it is plausible to postulate that circulating SARS-CoV-2 may interact with endothelial cells of hypertensive patients culminating both in direct viral injury and in a dysfunctional response to infection, amplificating chemokine release, inflammatory cell adhesion and migration through the endothelial barrier, ultimately leading to a procoagulant state and tissue damage (i.e., myocardial injury, acute respiratory distress syndrome) (Ackermann et al., 2020; Bermejo-Martin et al., 2020).

### Arterial Stiffness

Arterial stiffness describes the reduced capability of an artery to expand and contract in response to pressure changes. It is measured by carotid-femoral pulse wave velocity and is an independent predictor of cardiovascular (CV) events and mortality in patients with hypertension (Laurent et al., 2001).

Potentially, high arterial stiffness could have deleterious effects in patients with SARS-CoV-2 infection thorough different putative mechanisms: (i) chronically, arterial stiffness can increase the energy penetration of the increased pulsatile flow from the larger arteries, damaging target organs (brain, kidney, heart) and aggravating the infection by mitigating the functional reserves of different systems; (ii) the COVID-19 cytokine-storm may cause ventricular-arterial decoupling in the setting of low systemic vascular resistance and elevated heart rate. In this scenario, patients with high arterial stiffness could be more prone to ventricular-arterial decoupling by increasing pulsatile components of the total arterial load to the left ventricle (LV): proximal aortic impedance, wave reflections and arterial tree compliance (Chirinos et al., 2014, 2019; Ikonomidis et al., 2019) which ultimately leads to increased myocardial oxygen demand, CV inefficiency, and left ventricle (LV) energetic failure (Chemla et al., 2003; Guarracino et al., 2014); and (iii) increased arterial stiffness is associated with reduced coronary flow reserve in hypertensive patients (Ikonomidis et al., 2008) and lower diastolic blood pressure (coronary perfusion pressure), rendering these patients more vulnerable to myocardial injury and ischemia - a known complication of COVID-19 (Hendren et al., 2020).

Another intriguing aspect warranting exploration is whether survivors of COVID-19 will develop long term vascular sequelae of the infection, such as increased arterial stiffness and accelerated vascular aging. The Artery Society recently launched a collaborative, multicenter, research project to evaluate the vascular impact of the infection^1^ and will periodically test different biomarkers of aging in patients that had COVID-19.

### Left Ventricle Hypertrophy

Prolonged systemic hypertension results in hypertensive target-organ damage, and the most common manifestation of this is left ventricular hypertrophy (LVH). LVH - due to cellular hypertrophy and expansion of extracellular matrix - is defined as an increase in the mass of the left ventricle secondary to chronically elevated afterload and neurohormonal stimuli (Diez and Frohlich, 2010). Arterial stiffness plays a major role in LVH as it accelerates pulse wave velocity, causing premature arrival of wave reflections to the central aorta and by producing amplification of the mid-to-late systolic pressure, chronically stressing the LV (Chirinos et al., 2019). Allied to neurohormonal stimuli, both processes generate a series of molecular, cellular, and structural adaptations leading to cardiac remodeling (Nwabuo and Vasan, 2020). LVH is not only a marker of hypertension-related target organ damage but also an independent risk factor for CV complications (Haider et al., 1998; Sundstrom et al., 2001; Narayanan et al., 2014; Bang et al., 2017; Afify et al., 2018) that can occur after weaning of the initial compensatory mechanism due to contractile, electrical, structural or metabolic abnormalities (Pitoulis and Terracciano, 2020).

Myocardial injury among patients hospitalized with COVID-19 has been described since the early reports of the disease (Zhou et al., 2020a). Although being an important prognostic factor in severe cohorts (Guo et al., 2020; Lala et al., 2020; Shi et al., 2020a,b), the exact mechanism of myocardial injury is not fully understood as multiple plausible mechanisms often coexist in a single patient including multiorgan failure, types 1 and 2 myocardial infarction, disseminated intravascular coagulation, endothelial cell dysfunction, pre-existing chronic injury, pulmonary hypertension, among others (Jaffe et al., 2020). It is also unknown if there is a causal relationship between myocardial injury and disease severity or if it is solely a marker of pre-existing cardiovascular disease. LVH-related changes in the myocardial tissue and extracellular matrix might be related to both an increased risk of myocardial injury due to several pathophysiological pathways (increased cardiac Ang II, endothelial dysfunction, chronic inflammation, upregulated cardiac DPP4 expression) and an abolished cardiovascular response to the stress related to the infection.

Left ventricular hypertrophy imposed changes of cardiac structure function can complicate further the management of these patients in the intensive care unit (ICU), as LV compliance and diastolic function are impaired: the rise in the end-diastolic pressure narrows the optimal volume status for hemodynamic stability without pulmonary congestion (Sanfilippo et al., 2018); sinus tachycardia or supraventricular arrhythmias with rapid ventricular response can trigger hemodynamic collapse by reduced LV filling time-related to increase in the heart rate (Borlaug et al., 2011); increased LV filling pressure is also an independent risk factor for weaning failure from mechanical ventilation (Papanikolaou et al., 2011; Konomi et al., 2016; Liu et al., 2016) and the use high positive end-expiratory pressure during mechanical ventilation can lead to additional impairment in LV relaxation (Chin et al., 2013; Juhl-Olsen et al., 2013).

Also, the electrical remodeling due to LVH in hypertensive patients might be related to an increased risk of malignant ventricular arrhythmias and sudden cardiac death (Aro and Chugh, 2016), as they are being treated in the ICU with other aggravating factors such as mechanical ventilation, vasoactive drugs, medications that prolong the QT interval, and electrolyte disturbances. This overlap of hemodynamics and electrical disorders reflecting cellular and molecular remodeling due to LVH can indeed be the cause of poorer outcomes in patients with COVID-19 being treated in the ICU and poises a great challenge for clinicians that need to untangle the complexity of a serious illness aggravated by pre-existing conditions.

## Role of Dipeptidyl Peptidase 4 (DPP4)

Bioinformatic approaches based on protein crystal structure predicted that the middle east respiratory syndrome coronavirus (MERS-CoV) receptor DPP4 displays a high affinity with the SARS-CoV-2 spike protein (Li et al., 2020). This thereby suggests that SARS-CoV-2 may utilize DPP4 as a coreceptor, in addition to ACE2, to gain entry into the host cell. Nevertheless, the results of free energy calculation revealed that SARS-CoV-2 spike protein binds ACE2 with higher affinity than that of DPP4 (Li et al., 2020). Moreover, it was shown that only Hela and baby kidney hamster (BHK2) cells transfected with human ACE2, but not with human DPP4, were capable of being infected with SARS-CoV-2 (Hoffmann et al., 2020; Letko et al., 2020; Zhou et al., 2020b). However, further research is necessary to defined whether or not DPP4 may mediate the SARS-CoV-2 entry into permissive cells.

Dipeptidyl peptidase is a serine peptidase expressed on the surface of several cell types, including epithelial and endothelial cells and lymphocytes (Kenny et al., 1976; Marguet et al., 2000; Lambeir et al., 2003). It also exists as a soluble circulating form in plasma and other body fluids (Lambeir et al., 2003). Through its enzymatic function, DPP4 modulates the biological activity of several circulating hormones, neuropeptides, cytokines, and chemokines. In addition to its peptidase, activity DPP4 interacts with several proteins, including the renal proximal tubule Na^+^/H^+^ exchanger isoform 3 (NHE3) (Girardi et al., 2001), fibronectin and collagen, adenosine deaminase (ADA), C-X-C chemokine receptor type 4, underscoring the potential role of DPP4 in sodium retention, fibrosis, and inflammation. The importance of DPP4 for the scientific and medical community has considerably raised since the approval of inhibitors of DPP4 activity, known as gliptins, for the treatment of type 2 diabetes (T2D).

The gliptins do not bind to the putative receptor binding site of SARS-CoV-2 (Li et al., 2020). However, it does not exclude the possibility that DPP4 inhibition may indirectly attenuate the severity of COVID-19, due to the role that DPP4 plays in the pathophysiology of common comorbidities in patients with COVID-19 (Ryskjaer et al., 2006; Dos Santos et al., 2013; Zhong et al., 2013), including hypertension. Indeed, successive clinical studies have demonstrated that gliptins confer renal and cardiovascular benefits in patients with hypertension with or without T2D (Nistala and Savin, 2017). Intriguingly, although known primarily for its role as competitive inhibitors, gliptins are also capable of reducing DPP4 protein and mRNA abundance in the heart, kidneys, and endothelial cells of experimental animals of cardiovascular and metabolic diseases (Dos Santos et al., 2013; Kanasaki et al., 2014; Arruda-Junior et al., 2016; Beraldo et al., 2019). Whether altered DPP4 expression in the setting of hypertension, as well as of other comorbidities, contributes to SARS-CoV-2 infectivity, and COVID-19 severity is currently undetermined.

Recent evidence suggests the existence of an interplay between DPP4 and tissue RAS (Aroor et al., 2016; Beraldo et al., 2019). In renal proximal tubule cells, Ang II, through AT1R, enhances DPP4 activity, whereas inhibition of DPP4 mitigates Ang II-mediated activation of AT1R signaling and its downstream effects (Aroor et al., 2016). In rats with chronic kidney disease (CKD) and hypertension, the administration of the DPP4 inhibitor sitagliptin ameliorated hypertension, kidney function and restored the cardiac ratio of Ang II to Ang-(1-7) concentrations in the heart by reducing the levels of Ang II and increasing the content of Ang-(1-7) (Beraldo et al., 2019). Interestingly, sitagliptin was capable of upregulating ACE2 expression in the heart of rats with CKD and as well as in control animals (Beraldo et al., 2019). In line with these findings, Zhang et al. (2015) found that the DPP4 inhibitor linagliptin lowered the expression of the AT1R and upregulated the activity of ACE2 in the heart in rats with Ang II-induced hypertension. Collectively, these studies support the hypothesis that increased DPP4 activity and expression can favor an imbalance between ACE/Ang II/AT1R and ACE2/Ang-(1-7)/MasR.

The vascular activity and expression of DPP4 are increased in hypertensive rats (Linardi et al., 2004; Savignano et al., 2017), suggesting that this peptidase may contribute to impaired vascular function associated with high blood pressure. Accordingly, extensive studies have shown that DPP4 inhibitors play a protective effect against hypertension-related vascular events, such as endothelial dysfunction and increased arterial stiffness (Kishimoto et al., 2019; Zhang et al., 2019). The vasoprotective effects of DPP4 inhibition are mediated through multiple mechanisms, including improved NO bioavailability by upregulation of endothelial NOS, and thus, endothelium-dependent relaxation; reduction of ROS generation, and cyclooxygenase-2 expression; as well as by suppression of inflammatory responses (Zhang et al., 2019; Liu et al., 2020).

Several studies have demonstrated that DPP4 inhibitors ameliorate LVH (Dos Santos et al., 2013; Arruda-Junior et al., 2016; Beraldo et al., 2019; Nakajima et al., 2019; Nam et al., 2019; Okabe et al., 2020), whereas upregulated activity and expression of heart DPP4 is associated with cardiac remodeling and dysfunction (Dos Santos et al., 2013; Arruda-Junior et al., 2016; Beraldo et al., 2019). The antihypertrophic effects of the DPP4 inhibitor teneligliptin have been recently unraveled in an experimental model of Ang II-induced hypertension (Okabe et al., 2020). The authors found that the administration of teneligliptin to C57BL/6J mice suppressed Ang II-induced NADPH oxidase 4 mRNA overexpression, ROS production, and attenuated LVH without affecting blood pressure (Okabe et al., 2020). DPP4 inhibition has also mitigated LV remodeling and dysfunction in other experimental models of hypertension, such as spontaneously hypertensive rats and Dahl salt-sensitive rats (Nakajima et al., 2019; Nam et al., 2019). However, in the latter two studies, the gliptin-induced amelioration of cardiac remodeling and dysfunction was accompanied by blood pressure lowering effects.

## Concluding Remarks

**FIGURE 1:**
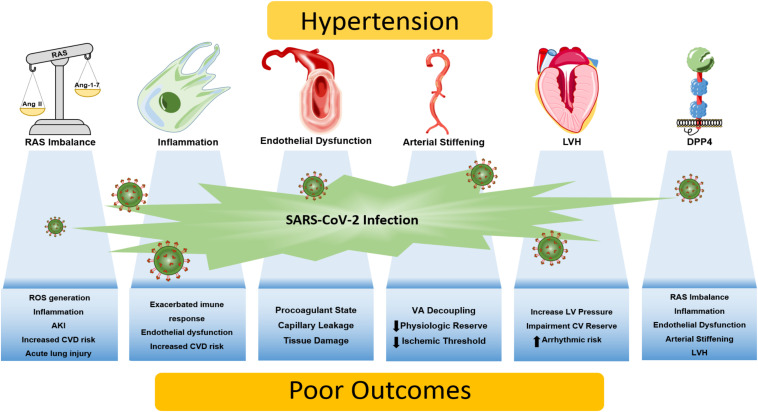
Putative mechanisms linking hypertension and COVID-19 severity. Patients with hypertension are more prone to a vicious interplay between RAS imbalance, chronic low-grade inflammation, and elevated DPP4 activity and expression. Dysregulation of these biological processes may be aggravated by the SARS-CoV-2 infection, giving rise to an exacerbated immune response that culminates in tissue damage/dysfunction. Also, end-organ damage caused by chronic hypertension diminishes cardiovascular reserve, as arterial stiffening, endothelial dysfunction and left ventricular hypertrophy emerges, leading to synergic processes that increase the susceptibility to know complications of COVID-19 including myocardial injury and ischemia, acute lung injury, thrombosis, acute kidney injury, ventricular arrhythmias and potentially death. AKI, acute kidney injury; CV, cardiovascular; CVD, cardiovascular disease; DPP4, dipeptidyl peptidase 4; LV, left ventricle; LVH, left ventricular hypertrophy; RAS, renin-angiotensin system; ROS, reactive oxygen species; VA, ventricle-atrial.

The relationship between hypertension, SARS-CoV-2 infection, and tissue injury is complex and multifactorial. Untangling the importance of several pathophysiological mechanisms in COVID-19 severity is still a work in progress, as scientific and clinical knowledge is continually being updated during the current pandemic. Through putative cellular, molecular and functional mechanisms, we provide a conceptual framework on how these biological processes may interact and lead to COVID-19 severity in patients with pre-existing hypertension: the role of the RAS, inflammation, endothelial dysfunction, arterial stiffness, left ventricular hypertrophy and DPP4 are summarized in Figure 1. In brief, patients with hypertension can be more prone to RAS imbalance, which in turn lead to vasoconstriction/inflammation due to unopposed Ang II effect, aggravated by increased DPP4 vascular activity/expression and by chronic low-grade inflammation. This dysregulated response, allied with diminished physiologic cardiovascular reserve induced by hypertension - arterial stiffening, left ventricular hypertrophy and endothelial dysfunction – creates the perfect milieu for both COVID-19 related tissue injury and worsening of cardiac, renal and vascular function.

Targeting these biological processes might attenuate the inflammatory response, reduce tissue injury, and ultimately lead to better outcomes in hypertensive patients with SARS-CoV-2 infection. Also, understanding the pathophysiology of hypertension in cardiovascular hemodynamics and how it might lead to poor outcomes in COVID-19 patients can aid the clinician in making decisions at the bedside. Finally, the role of RAS inhibitors needs to be further investigated, but, to the best of our knowledge, there is no known harmful impact of these medications neither on the risk of infection or disease severity. Noteworthy, preliminary data suggest that these antihypertensive agents may, in fact, confer a protective effect.

## Author Contributions

All authors conceived, wrote the manuscript and contributed to the article and approved the submitted version.

## Conflict of Interest

The authors declare that the research was conducted in the absence of any commercial or financial relationships that could be construed as a potential conflict of interest.

## Footnotes

1 http://www.arterysociety.org/our-activities/cartesian-2/

## References

[B1] Abais-Battad J. M. Lund H. Fehrenbach D. J. Dasinger J. H. Mattson D. L. (2018). Rag1-null Dahl SS rats reveal that adaptive immune mechanisms exacerbate high protein-induced hypertension and renal injury. *Am. J. Physiol. Regul. Integr. Comp. Physiol.* 315 R28–R35. 29537860 10.1152/ajpregu.00201.2017 PMC6087888

[B2] Ackermann M. Verleden S. E. Kuehnel M. Haverich A. Welte T. Laenger F. (2020). Pulmonary vascular endothelialitis, thrombosis, and angiogenesis in Covid-19. *N. Engl. J. Med.* 383 120–128. 10.1056/nejmoa2015432 32437596 PMC7412750

[B3] Afify H. M. A. Waits G. S. Ghoneum A. D. Cao X. Li Y. Soliman E. Z. (2018). Peguero electrocardiographic left ventricular hypertrophy criteria and risk of mortality. *Front. Cardiovasc. Med.* 5:75. 10.3389/fcvm.2018.00075 30013976 PMC6036297

[B4] Ainscough J. F. Drinkhill M. J. Sedo A. Turner N. A. Brooke D. A. Balmforth A. J. (2009). Angiotensin II type-1 receptor activation in the adult heart causes blood pressure-independent hypertrophy and cardiac dysfunction. *Cardiovasc. Res.* 81 592–600. 10.1093/cvr/cvn230 18703536

[B5] Aro A. L. Chugh S. S. (2016). Clinical diagnosis of electrical versus anatomic left ventricular hypertrophy: prognostic and therapeutic implications. *Circ. Arrhythm. Electrophysiol.* 9:e003629. 10.1161/CIRCEP.115.003629 PMC4807859 27009417

[B6] Aroor A. Zuberek M. Duta C. Meuth A. Sowers J. R. Whaley-Connell A. (2016). Angiotensin II stimulation of DPP4 activity regulates megalin in the proximal tubules. *Int. J. Mol. Sci.* 17:780. 10.3390/ijms17050780 27213360 PMC4881597

[B7] Arruda-Junior D. F. Martins F. L. Dariolli R. Jensen L. Antonio E. L. Dos Santos L. (2016). Dipeptidyl peptidase IV inhibition exerts renoprotective effects in rats with established heart failure. *Front. Physiol.* 7:293. 10.3389/fphys.2016.00293 27462276 PMC4941796

[B8] Bang C. N. Soliman E. Z. Simpson L. M. Davis B. R. Devereux R. B. Okin P. M. (2017). Electrocardiographic left ventricular hypertrophy predicts cardiovascular morbidity and mortality in hypertensive patients: the ALLHAT study. *Am. J. Hypertens.* 30 914–922. 10.1093/ajh/hpx067 28430947 PMC5861536

[B9] Benigni A. Cassis P. Remuzzi G. (2010). Angiotensin II revisited: new roles in inflammation, immunology and aging. *EMBO Mol. Med.* 2 247–257. 10.1002/emmm.201000080 20597104 PMC3377325

[B10] Beraldo J. I. Benetti A. Borges-Júnior F. A. Arruda-Junior D. F. Martins F. L. Jensen L. (2019). Cardioprotection conferred by sitagliptin is associated with reduced cardiac angiotensin II/Angiotensin-(1-7) balance in experimental chronic kidney disease. *Int. J. Mol. Sci.* 20:1940. 10.3390/ijms20081940 31010001 PMC6515057

[B11] Bermejo-Martin J. F. Almansa R. Torres A. Gonzalez-Rivera M. Kelvin D. J. (2020). COVID-19 as a cardiovascular disease: the potential role of chronic endothelial dysfunction. *Cardiovasc. Res.* 116 e132–e133. 32420587 10.1093/cvr/cvaa140 PMC7314234

[B12] Borlaug B. A. Jaber W. A. Ommen S. R. Lam C. S. Redfield M. M. Nishimura R. A. (2011). Diastolic relaxation and compliance reserve during dynamic exercise in heart failure with preserved ejection fraction. *Heart* 97 964–969. 10.1136/hrt.2010.212787 21478380 PMC3767403

[B13] Braun F. Lütgehetmann M. Pfefferle S. Wong M. N. Carsten A. Lindenmeyer M. T. (2020). SARS-CoV-2 renal tropism associates with acute kidney injury. *Lancet* 396 597–598. 10.1016/s0140-6736(20)31759-1 32818439 PMC7431179

[B14] Carson P. Giles T. Higginbotham M. Hollenberg N. Kannel W. Siragy H. M. (2001). Angiotensin receptor blockers: evidence for preserving target organs. *Clin. Cardiol.* 24 183–190. 10.1002/clc.4960240303 11288962 PMC6654811

[B15] Chemla D. Antony I. Lecarpentier Y. Nitenberg A. (2003). Contribution of systemic vascular resistance and total arterial compliance to effective arterial elastance in humans. *Am. J. Physiol. Heart Circ. Physiol.* 285 H614–H620. 12689857 10.1152/ajpheart.00823.2002

[B16] Cheng Y. Luo R. Wang K. Zhang M. Wang Z. Dong L. (2020). Kidney disease is associated with in-hospital death of patients with COVID-19. *Kidney Int.* 97 829–838. 10.1016/j.kint.2020.03.005 32247631 PMC7110296

[B17] Chin J. H. Lee E. H. Kim W. J. Choi D. K. Hahm K. D. Sim J. Y. (2013). Positive end-expiratory pressure aggravates left ventricular diastolic relaxation further in patients with pre-existing relaxation abnormality. *Br. J. Anaesth.* 111 368–373. 10.1093/bja/aet061 23533256

[B18] Chirinos J. A. Rietzschel E. R. Shiva-Kumar P. De Buyzere M. L. Zamani P. Claessens T. (2014). Effective arterial elastance is insensitive to pulsatile arterial load. *Hypertension* 64 1022–1031. 10.1161/hypertensionaha.114.03696 25069668

[B19] Chirinos J. A. Segers P. Hughes T. Townsend R. (2019). Large-artery stiffness in health and disease: JACC state-of-the-art review. *J. Am. Coll. Cardiol.* 74 1237–1263. 10.1016/j.jacc.2019.07.012 31466622 PMC6719727

[B20] Crackower M. A. Sarao R. Oudit G. Y. Yagil C. Kozieradzki I. Scanga S. E. (2002). Angiotensin-converting enzyme 2 is an essential regulator of heart function. *Nature* 417 822–828. 12075344 10.1038/nature00786

[B21] Crowley S. D. Gurley S. B. Herrera M. J. Ruiz P. Griffiths R. Kumar A. P. (2006). Angiotensin II causes hypertension and cardiac hypertrophy through its receptors in the kidney. *Proc. Natl. Acad. Sci. U.S.A.* 103 17985–17990. 10.1073/pnas.0605545103 17090678 PMC1693859

[B22] Crowley S. D. Gurley S. B. Oliverio M. I. Pazmino A. K. Griffiths R. Flannery P. J. (2005). Distinct roles for the kidney and systemic tissues in blood pressure regulation by the renin-angiotensin system. *J. Clin. Invest.* 115 1092–1099. 10.1172/jci23378 15841186 PMC1070417

[B23] Czopek A. Moorhouse R. Guyonnet L. Farrah T. Lenoir O. Owen E. (2019). A novel role for myeloid endothelin-B receptors in hypertension. *Eur. Heart J.* 40 768–784. 10.1093/eurheartj/ehy881 30657897 PMC6396028

[B24] Dahlof B. Devereux R. B. Kjeldsen S. E. Julius S. Beevers G. De Faire U. (2002). Cardiovascular morbidity and mortality in the Losartan Intervention For Endpoint reduction in hypertension study (LIFE): a randomised trial against atenolol. *Lancet* 359 995–1003. 10.1016/s0140-6736(02)08089-3 11937178

[B25] Deanfield J. E. Halcox J. P. Rabelink T. J. (2007). Endothelial function and dysfunction: testing and clinical relevance. *Circulation* 115 1285–1295. 10.1161/circulationaha.106.652859 17353456

[B26] Diaz J. H. (2020). Hypothesis: angiotensin-converting enzyme inhibitors and angiotensin receptor blockers may increase the risk of severe COVID-19. *J. Travel Med.* 27:taaa041. 10.1093/jtm/taaa041 PMC7184445 32186711

[B27] Diez J. Frohlich E. D. (2010). A translational approach to hypertensive heart disease. *Hypertension* 55 1–8. 10.1161/hypertensionaha.109.141887 19933923

[B28] Dong E. Du H. Gardner L. (2020). An interactive web-based dashboard to track COVID-19 in real time. *Lancet Infect. Dis.* 20 533–534. 10.1016/s1473-3099(20)30120-1 32087114 PMC7159018

[B29] Donoghue M. Hsieh F. Baronas E. Godbout K. Gosselin M. Stagliano N. (2000). A novel angiotensin-converting enzyme-related carboxypeptidase (ACE2) converts angiotensin I to angiotensin 1-9. *Circ. Res.* 87 E1–E9. 10969042 10.1161/01.res.87.5.e1

[B30] Dos Santos L. Salles T. A. Arruda-Junior D. F. Campos L. C. G. Pereira A. C. Barreto A. L. T. (2013). Circulating dipeptidyl peptidase IV activity correlates with cardiac dysfunction in human and experimental heart failure. *Circulation* 6 1029–1038. 10.1161/circheartfailure.112.000057 23894014

[B31] Esler M. Esler D. (2020). Can angiotensin receptor-blocking drugs perhaps be harmful in the COVID-19 pandemic? *J. Hypertens* 38 781–782. 10.1097/hjh.0000000000002450 32195824

[B32] Fehrenbach D. J. Dasinger J. H. Lund H. Zemaj J. Mattson D. L. (2020). Splenocyte transfer exacerbates salt-sensitive hypertension in rats. *Exp. Physiol.* 105 864–875. 10.1113/ep088340 32034948 PMC7864544

[B33] Ferrario C. M. Jessup J. Chappell M. C. Averill D. B. Brosnihan K. B. Tallant E. A. (2005). Effect of angiotensin-converting enzyme inhibition and angiotensin II receptor blockers on cardiac angiotensin-converting enzyme 2. *Circulation* 111 2605–2610. 10.1161/circulationaha.104.510461 15897343

[B34] Garg S. Kim L. Whitaker M. O’halloran A. Cummings C. Holstein R. (2020). Hospitalization rates and characteristics of patients hospitalized with laboratory-confirmed coronavirus disease 2019 – COVID-NET, 14 States, March 1-30, 2020. *MMWR Morb. Mortal. Wkly. Rep.* 69 458–464. 10.15585/mmwr.mm6915e3 32298251 PMC7755063

[B35] Gembardt F. Sterner-Kock A. Imboden H. Spalteholz M. Reibitz F. Schultheiss H. P. (2005). Organ-specific distribution of ACE2 mRNA and correlating peptidase activity in rodents. *Peptides* 26 1270–1277. 10.1016/j.peptides.2005.01.009 15949646 PMC7115528

[B36] Girardi A. C. C. Degray B. C. Nagy T. Biemesderfer D. Aronson P. S. (2001). Association of Na+-H+ exchanger isoform NHE3 and dipeptidyl peptidase IV in the renal proximal tubule. *J. Biol. Chem.* 276 46671–46677. 10.1074/jbc.m106897200 11590171

[B37] Grasselli G. Zangrillo A. Zanella A. Antonelli M. Cabrini L. Castelli A. (2020). Baseline characteristics and outcomes of 1591 patients infected with SARS-CoV-2 admitted to ICUs of the Lombardy Region, Italy. *JAMA* 323 1574–1581. 10.1001/jama.2020.5394 32250385 PMC7136855

[B38] Guan W. J. Liang W. H. Zhao Y. Liang H. R. Chen Z. S. Li Y. M. (2020a). Comorbidity and its impact on 1590 patients with COVID-19 in China: a nationwide analysis. *Eur. Respir. J.* 55:2000547. 10.1183/13993003.00547-2020 PMC7098485 32217650

[B39] Guan W. J. Ni Z. Y. Hu Y. Liang W. H. Ou C. Q. He J. X. (2020b). Clinical characteristics of coronavirus disease 2019 in China. *N. Engl. J. Med.* 382 1708–1720. 32109013 10.1056/NEJMoa2002032 PMC7092819

[B40] Guarracino F. Ferro B. Morelli A. Bertini P. Baldassarri R. Pinsky M. R. (2014). Ventriculoarterial decoupling in human septic shock. *Crit. Care* 18 R80. 10.1186/cc13842 PMC4056562 24762124

[B41] Guo T. Fan Y. Chen M. Wu X. Zhang L. He T. (2020). Cardiovascular implications of fatal outcomes of patients with coronavirus disease 2019 (COVID-19). *JAMA Cardiol.* 5 811–818. 10.1001/jamacardio.2020.1017 32219356 PMC7101506

[B42] Gurley S. B. Allred A. Le T. H. Griffiths R. Mao L. Philip N. (2006). Altered blood pressure responses and normal cardiac phenotype in ACE2-null mice. *J. Clin. Invest.* 116 2218–2225. 10.1172/jci16980 16878172 PMC1518789

[B43] Gurley S. B. Riquier-Brison A. D. M. Schnermann J. Sparks M. A. Allen A. M. Haase V. H. (2011). AT1A angiotensin receptors in the renal proximal tubule regulate blood pressure. *Cell Metab.* 13 469–475. 10.1016/j.cmet.2011.03.001 21459331 PMC3070917

[B44] Guyonnet L. Czopek A. Farrah T. E. Baudrie V. Bonnin P. Chipont A. (2020). Deletion of the myeloid endothelin-B receptor confers long-term protection from angiotensin II-mediated kidney, eye and vessel injury. *Kidney Int.* 98 1193–1209. 10.1016/j.kint.2020.05.042 32569653 PMC7652550

[B45] Guzik T. J. Hoch N. E. Brown K. A. Mccann L. A. Rahman A. Dikalov S. (2007). Role of the T cell in the genesis of angiotensin II induced hypertension and vascular dysfunction. *J. Exp. Med.* 204 2449–2460. 10.1084/jem.20070657 17875676 PMC2118469

[B46] Haider A. W. Larson M. G. Benjamin E. J. Levy D. (1998). Increased left ventricular mass and hypertrophy are associated with increased risk for sudden death. *J. Am. Coll. Cardiol.* 32 1454–1459. 10.1016/s0735-1097(98)00407-0 9809962

[B47] Hendren N. S. Drazner M. H. Bozkurt B. Cooper L. T. Jr. (2020). Description and proposed management of the acute COVID-19 cardiovascular syndrome. *Circulation* 141 1903–1914. 10.1161/circulationaha.120.047349 32297796 PMC7314493

[B48] Heurich A. Hofmann-Winkler H. Gierer S. Liepold T. Jahn O. Pohlmann S. (2014). TMPRSS2 and ADAM17 cleave ACE2 differentially and only proteolysis by TMPRSS2 augments entry driven by the severe acute respiratory syndrome coronavirus spike protein. *J. Virol.* 88 1293–1307. 10.1128/jvi.02202-13 24227843 PMC3911672

[B49] Hingorani A. D. Cross J. Kharbanda R. K. Mullen M. J. Bhagat K. Taylor M. (2000). Acute systemic inflammation impairs endothelium-dependent dilatation in humans. *Circulation* 102 994–999. 10.1161/01.cir.102.9.994 10961963

[B50] Hoffmann M. Kleine-Weber H. Schroeder S. Kruger N. Herrler T. Erichsen S. (2020). SARS-CoV-2 cell entry depends on ACE2 and TMPRSS2 and is blocked by a clinically proven protease inhibitor. *Cell* 181 271–280.e8. 32142651 10.1016/j.cell.2020.02.052 PMC7102627

[B51] Huang C. Wang Y. Li X. Ren L. Zhao J. Hu Y. (2020). Clinical features of patients infected with 2019 novel coronavirus in Wuhan, China. *Lancet* 395 497–506. 31986264 10.1016/S0140-6736(20)30183-5 PMC7159299

[B52] Huentelman M. J. Grobe J. L. Vazquez J. Stewart J. M. Mecca A. P. Katovich M. J. (2005). Protection from angiotensin II-induced cardiac hypertrophy and fibrosis by systemic lentiviral delivery of ACE2 in rats. *Exp. Physiol.* 90 783–790. 10.1113/expphysiol.2005.031096 16049057

[B53] Ikonomidis I. Aboyans V. Blacher J. Brodmann M. Brutsaert D. L. Chirinos J. A. (2019). The role of ventricular-arterial coupling in cardiac disease and heart failure: assessment, clinical implications and therapeutic interventions. A consensus document of the European society of cardiology working group on aorta & peripheral vascular diseases, European association of cardiovascular imaging, and heart failure association. *Eur. J. Heart Fail.* 21 402–424. 10.1002/ejhf.1436 30859669

[B54] Ikonomidis I. Lekakis J. Papadopoulos C. Triantafyllidi H. Paraskevaidis I. Georgoula G. (2008). Incremental value of pulse wave velocity in the determination of coronary microcirculatory dysfunction in never-treated patients with essential hypertension. *Am. J. Hypertens.* 21 806–813. 10.1038/ajh.2008.172 18497732

[B55] Imai Y. Kuba K. Rao S. Huan Y. Guo F. Guan B. (2005). Angiotensin-converting enzyme 2 protects from severe acute lung failure. *Nature* 436 112–116. 10.1038/nature03712 16001071 PMC7094998

[B56] Itani H. A. Mcmaster W. G. Saleh M. A. Nazarewicz R. R. Mikolajczyk T. P. Kaszuba A. M. (2016). Activation of human T cells in hypertension: studies of humanized mice and hypertensive humans. *Hypertension* 68 123–132. 10.1161/hypertensionaha.116.07237 27217403 PMC4900908

[B57] Iwata-Yoshikawa N. Okamura T. Shimizu Y. Hasegawa H. Takeda M. Nagata N. (2019). TMPRSS2 contributes to virus spread and immunopathology in the airways of murine models after coronavirus infection. *J. Virol.* 93 e01815-18. 10.1128/JVI.01815-18 30626688 PMC6401451

[B58] Jaffe A. S. Cleland J. G. F. Katus H. A. (2020). Myocardial injury in severe COVID-19 infection. *Eur. Heart J.* 41 2080–2082. 10.1093/eurheartj/ehaa447 32464642 PMC7314085

[B59] Jessup J. A. Gallagher P. E. Averill D. B. Brosnihan K. B. Tallant E. A. Chappell M. C. (2006). Effect of angiotensin II blockade on a new congenic model of hypertension derived from transgenic Ren-2 rats. *Am. J. Physiol. Heart Circ. Physiol.* 291 H2166–H2172. 16766648 10.1152/ajpheart.00061.2006

[B60] Juhl-Olsen P. Hermansen J. F. Frederiksen C. A. Rasmussen L. A. Jakobsen C. J. Sloth E. (2013). Positive end-expiratory pressure influences echocardiographic measures of diastolic function: a randomized, crossover study in cardiac surgery patients. *Anesthesiology* 119 1078–1086. 10.1097/aln.0b013e3182a10b40 23823106

[B61] Kanasaki K. Shi S. Kanasaki M. He J. Nagai T. Nakamura Y. (2014). Linagliptin-mediated DPP-4 inhibition ameliorates kidney fibrosis in streptozotocin-induced diabetic mice by inhibiting endothelial-to-mesenchymal transition in a therapeutic regimen. *Diabetes Metab. Res. Rev* 63 2120–2131. 10.2337/db13-1029 24574044

[B62] Kenny A. J. Booth A. G. George S. G. Ingram J. Kershaw D. Wood E. J. (1976). Dipeptidyl peptidase IV, a kidney brush-border serine peptidase. *Biochem. J.* 157 169–182. 10.1042/bj1570169 962853 PMC1163828

[B63] Kishimoto S. Kinoshita Y. Matsumoto T. Maruhashi T. Kajikawa M. Matsui S. (2019). Effects of the dipeptidyl peptidase 4 inhibitor alogliptin on blood pressure in hypertensive patients with type 2 diabetes mellitus. *Am. J. Hypertens.* 32 695–702. 10.1093/ajh/hpz065 31045223

[B64] Koka V. Huang X. R. Chung A. C. Wang W. Truong L. D. Lan H. Y. (2008). Angiotensin II up-regulates angiotensin I-converting enzyme (ACE), but down-regulates ACE2 via the AT1-ERK/p38 MAP kinase pathway. *Am. J. Pathol.* 172 1174–1183. 10.2353/ajpath.2008.070762 18403595 PMC2329828

[B65] Konomi I. Tasoulis A. Kaltsi I. Karatzanos E. Vasileiadis I. Temperikidis P. (2016). Left ventricular diastolic dysfunction–an independent risk factor for weaning failure from mechanical ventilation. *Anaesth. Intensive Care* 44 466–473. 10.1177/0310057x1604400408 27456176

[B66] Kuba K. Imai Y. Rao S. Gao H. Guo F. Guan B. (2005). A crucial role of angiotensin converting enzyme 2 (ACE2) in SARS coronavirus-induced lung injury. *Nat. Med.* 11 875–879. 10.1038/nm1267 16007097 PMC7095783

[B67] Lala A. Johnson K. W. Januzzi J. L. Russak A. J. Paranjpe I. Richter F. (2020). Prevalence and impact of myocardial injury in patients hospitalized with COVID-19 infection. *J. Am. Coll. Cardiol.* 76 533–546. 32517963 10.1016/j.jacc.2020.06.007 PMC7279721

[B68] Lambeir A. M. Durinx C. Scharpé S. De Meester I. (2003). Dipeptidyl-peptidase IV from bench to bedside: an update on structural properties, functions, and clinical aspects of the enzyme DPP IV. *Crit. Rev. Clin. Lab. Sci* 40 209–294. 10.1080/713609354 12892317

[B69] Laurent S. Boutouyrie P. Asmar R. Gautier I. Laloux B. Guize L. (2001). Aortic stiffness is an independent predictor of all-cause and cardiovascular mortality in hypertensive patients. *Hypertension* 37 1236–1241. 10.1161/01.hyp.37.5.1236 11358934

[B70] Letko M. Marzi A. Munster V. (2020). Functional assessment of cell entry and receptor usage for SARS-CoV-2 and other lineage B betacoronaviruses. *Nat. Microbiol.* 5 562–569. 10.1038/s41564-020-0688-y 32094589 PMC7095430

[B71] Li Y. Zhang Z. Yang L. Lian X. Xie Y. Li S. (2020). The MERS-CoV receptor DPP4 as a candidate binding target of the SARS-CoV-2 Spike. *iScience* 23:101160 10.1016/j.isci.2020.101160 PMC7219414 32405622

[B72] Linardi A. Panunto P. C. Ferro E. S. Hyslop S. (2004). Peptidase activities in rats treated chronically with N(omega)-nitro-L-arginine methyl ester (L-NAME). *Biochem. Pharmacol.* 68 205–214. 10.1016/j.bcp.2004.03.016 15193992

[B73] Liu H. Guo L. Xing J. Li P. Sang H. Hu X. (2020). The protective role of DPP4 inhibitors in atherosclerosis. *Eur. J. Pharmacol.* 875:173037 10.1016/j.ejphar.2020.173037 32097656

[B74] Liu J. Shen F. Teboul J. L. Anguel N. Beurton A. Bezaz N. (2016). Cardiac dysfunction induced by weaning from mechanical ventilation: incidence, risk factors, and effects of fluid removal. *Crit. Care* 20:369. 10.1186/s13054-016-1533-9 PMC5106814 27836002

[B75] Machnik A. Neuhofer W. Jantsch J. Dahlmann A. Tammela T. Machura K. (2009). Macrophages regulate salt-dependent volume and blood pressure by a vascular endothelial growth factor-C-dependent buffering mechanism. *Nat. Med.* 15 545–552. 10.1038/nm.1960 19412173

[B76] Mancia G. Rea F. Ludergnani M. Apolone G. Corrao G. (2020). Renin-angiotensin-aldosterone system blockers and the risk of Covid-19. *N. Engl. J. Med.* 382 2431–2440. 10.1056/nejmoa2006923 32356627 PMC7206933

[B77] Marguet D. Baggio L. Kobayashi T. Bernard A. M. Pierres M. Nielsen P. F. (2000). Enhanced insulin secretion and improved glucose tolerance in mice lacking CD26. *Proc. Natl. Acad. Sci. U.S.A.* 97 6874–6879. 10.1073/pnas.120069197 10823914 PMC18768

[B78] Mattson D. L. (2019). Immune mechanisms of salt-sensitive hypertension and renal end-organ damage. *Nat. Rev. Nephrol.* 15 290–300. 10.1038/s41581-019-0121-z 30804523

[B79] Mehta N. Kalra A. Nowacki A. S. Anjewierden S. Han Z. Bhat P. (2020). Association of use of angiotensin-converting enzyme inhibitors and angiotensin II receptor blockers with testing positive for coronavirus disease 2019 (COVID-19). *JAMA Cardiol.* 5 1020–1026. 10.1001/jamacardio.2020.1855 32936273 PMC7201375

[B80] Nakajima Y. Ito S. Asakura M. Min K. D. Fu H. Y. Imazu M. (2019). A dipeptidyl peptidase-IV inhibitor improves diastolic dysfunction in Dahl salt-sensitive rats. *J. Mol. Cell Cardiol.* 129 257–265. 10.1016/j.yjmcc.2019.03.009 30880253

[B81] Nam D. H. Park J. Park S. H. Kim K. S. Baek E. B. (2019). Effect of gemigliptin on cardiac ischemia/reperfusion and spontaneous hypertensive rat models. *Korean J. Physiol. Pharmacol.* 23 329–334. 31496870 10.4196/kjpp.2019.23.5.329 PMC6717789

[B82] Narayanan K. Reinier K. Teodorescu C. Uy-Evanado A. Chugh H. Gunson K. (2014). Electrocardiographic versus echocardiographic left ventricular hypertrophy and sudden cardiac arrest in the community. *Heart Rhythm* 11 1040–1046. 10.1016/j.hrthm.2014.03.023 24657425 PMC4035427

[B83] Nistala R. Savin V. (2017). Diabetes, hypertension, and chronic kidney disease progression: role of DPP4. *Am. J. Physiol. Renal Physiol.* 312 F661–F670. 28122713 10.1152/ajprenal.00316.2016

[B84] Nwabuo C. C. Vasan R. S. (2020). Pathophysiology of hypertensive heart disease: beyond left ventricular hypertrophy. *Curr. Hypertens. Rep* 22:11. 10.1007/s11906-020-1017-9 32016791

[B85] Okabe K. Matsushima S. Ikeda S. Ikeda M. Ishikita A. Tadokoro T. (2020). DPP (Dipeptidyl Peptidase)-4 inhibitor attenuates Ang II (Angiotensin II)-induced cardiac hypertrophy via GLP (Glucagon-Like Peptide)-1-Dependent Suppression of Nox (Nicotinamide Adenine Dinucleotide Phosphate Oxidase) 4-HDAC (Histone Deacetylase) 4 Pathway. *Hypertension* 75 991–1001. 10.1161/hypertensionaha.119.14400 32160098

[B86] Papanikolaou J. Makris D. Saranteas T. Karakitsos D. Zintzaras E. Karabinis A. (2011). New insights into weaning from mechanical ventilation: left ventricular diastolic dysfunction is a key player. *Intensive Care Med.* 37 1976–1985. 10.1007/s00134-011-2368-0 21976188

[B87] Pinto B. G. G. Oliveira A. E. R. Singh Y. Jimenez L. Gonçalves A. N. A. Ogava R. L. T. (2020). ACE2 expression is increased in the lungs of patients with comorbidities associated with severe COVID-19. *J. Infect. Dis.* 222 556–563. 10.1093/infdis/jiaa332 32526012 PMC7377288

[B88] Pitoulis F. G. Terracciano C. M. (2020). Heart plasticity in response to pressure- and volume-overload: a review of findings in compensated and decompensated phenotypes. *Front. Physiol.* 11:92. 10.3389/fphys.2020.00092 32116796 PMC7031419

[B89] Puelles V. G. Lütgehetmann M. Lindenmeyer M. T. Sperhake J. P. Wong M. N. Allweiss L. (2020). Multiorgan and Renal Tropism of SARS-CoV-2. *N. Engl. J. Med.* 383 590–592. 10.1056/nejmc2011400 32402155 PMC7240771

[B90] Reynolds H. R. Adhikari S. Pulgarin C. Troxel A. B. Iturrate E. Johnson S. B. (2020). Renin-Angiotensin-Aldosterone System Inhibitors and Risk of Covid-19. *N. Engl. J. Med.* 382 2441–2448. 32356628 10.1056/NEJMoa2008975 PMC7206932

[B91] Richardson S. Hirsch J. S. Narasimhan M. Crawford J. M. Mcginn T. Davidson K. W. (2020). Presenting characteristics, comorbidities, and outcomes among 5700 patients hospitalized with COVID-19 in the New York City Area. *JAMA* 323 2052–2059. 32320003 10.1001/jama.2020.6775 PMC7177629

[B92] Rosenblat M. Elias A. Volkova N. Aviram M. (2013). Monocyte-macrophage membrane possesses free radicals scavenging activity: stimulation by polyphenols or by paraoxonase 1 (PON1). *Free Radic. Res.* 47 257–267. 10.3109/10715762.2013.765562 23316782

[B93] Ryskjaer J. Deacon C. F. Carr R. D. Krarup T. Madsbad S. Holst J. (2006). Plasma dipeptidyl peptidase-IV activity in patients with type-2 diabetes mellitus correlates positively with HbAlc levels, but is not acutely affected by food intake. *Eur. J. Endocrinol.* 155 485–493. 10.1530/eje.1.02221 16914604

[B94] Sadoshima J. Izumo S. (1993). Molecular characterization of angiotensin II–induced hypertrophy of cardiac myocytes and hyperplasia of cardiac fibroblasts. *Critical role of the AT*1 receptor subtype. *Circ. Res.* 73 413–423. 10.1161/01.res.73.3.413 8348686

[B95] Sampaio W. O. Henrique De Castro C. Santos R. A. Schiffrin E. L. Touyz R. M. (2007). Angiotensin-(1-7) counterregulates angiotensin II signaling in human endothelial cells. *Hypertension* 50 1093–1098. 10.1161/hypertensionaha.106.084848 17984366

[B96] Sanfilippo F. Scolletta S. Morelli A. Vieillard-Baron A. (2018). Practical approach to diastolic dysfunction in light of the new guidelines and clinical applications in the operating room and in the intensive care. *Ann. Intensive Care* 8 100. 10.1186/s13613-018-0447-x PMC6206316 30374644

[B97] Santos R. A. S. Sampaio W. O. Alzamora A. C. Motta-Santos D. Alenina N. Bader M. (2018). The ACE2/Angiotensin-(1-7)/MAS axis of the renin-angiotensin system: focus on angiotensin-(1-7). *Physiol. Rev.* 98 505–553. 10.1152/physrev.00023.2016 29351514 PMC7203574

[B98] Savignano F. A. Crajoinas R. O. Pacheco B. P. M. Campos L. C. G. Shimizu M. H. M. Seguro A. C. (2017). Attenuated diuresis and natriuresis in response to glucagon-like peptide-1 in hypertensive rats are associated with lower expression of the glucagon-like peptide-1 receptor in the renal vasculature. *Eur. J. Pharmacol* 811 38–47. 10.1016/j.ejphar.2017.05.054 28576404

[B99] Seniuk A. Thiele J. L. Stubbe A. Oser P. Rosendahl A. Bode M. (2020). B6.R*ag*1 knockout mice generated at the Jackson Laboratory in 2009 show a robust wild-type hypertensive phenotype in response to Ang II (Angiotensin II). *Hypertension* 75 1110–1116. 10.1161/hypertensionaha.119.13773 32078412

[B100] Shi S. Qin M. Cai Y. Liu T. Shen B. Yang F. (2020a). Characteristics and clinical significance of myocardial injury in patients with severe coronavirus disease 2019. *Eur. Heart J.* 41 2070–2079. 10.1093/eurheartj/ehaa408 32391877 PMC7239100

[B101] Shi S. Qin M. Shen B. Cai Y. Liu T. Yang F. (2020b). Association of cardiac injury with mortality in hospitalized patients with COVID-19 in Wuhan, China. *JAMA Cardiol.* 5 802–810. 10.1001/jamacardio.2020.0950 32211816 PMC7097841

[B102] Shimada S. Abais-Battad J. M. Alsheikh A. J. Yang C. Stumpf M. Kurth T. (2020). Renal perfusion pressure determines infiltration of leukocytes in the kidney of rats with angiotensin II-induced hypertension. *Hypertension* 76 849–858. 10.1161/hypertensionaha.120.15295 32755400 PMC7429333

[B103] Soler M. J. Wysocki J. Batlle D. (2013). ACE2 alterations in kidney disease. *Nephrol. Dial. Transplant.* 28 2687–2697. 10.1093/ndt/gft320 23956234 PMC3811059

[B104] Sundstrom J. Lind L. Arnlov J. Zethelius B. Andren B. Lithell H. O. (2001). Echocardiographic and electrocardiographic diagnoses of left ventricular hypertrophy predict mortality independently of each other in a population of elderly men. *Circulation* 103 2346–2351. 10.1161/01.cir.103.19.2346 11352882

[B105] Tang N. Li D. Wang X. Sun Z. (2020). Abnormal coagulation parameters are associated with poor prognosis in patients with novel coronavirus pneumonia. *J. Thromb. Haemost.* 18 844–847. 10.1111/jth.14768 32073213 PMC7166509

[B106] Tipnis S. R. Hooper N. M. Hyde R. Karran E. Christie G. Turner A. J. (2000). A human homolog of angiotensin-converting enzyme. Cloning and functional expression as a captopril-insensitive carboxypeptidase. *J. Biol. Chem.* 275 33238–33243. 10.1074/jbc.m002615200 10924499

[B107] Varga Z. Flammer A. J. Steiger P. Haberecker M. Andermatt R. Zinkernagel A. S. (2020). Endothelial cell infection and endotheliitis in COVID-19. *Lancet* 395 1417–1418. 32325026 10.1016/S0140-6736(20)30937-5 PMC7172722

[B108] Wakahara S. Konoshita T. Mizuno S. Motomura M. Aoyama C. Makino Y. (2007). Synergistic expression of angiotensin-converting enzyme (ACE) and ACE2 in human renal tissue and confounding effects of hypertension on the ACE to ACE2 ratio. *Endocrinology* 148 2453–2457. 17303661 10.1210/en.2006-1287

[B109] Wang D. Hu B. Hu C. Zhu F. Liu X. Zhang J. (2020). Clinical characteristics of 138 hospitalized patients with 2019 novel coronavirus-infected pneumonia in Wuhan, China. *JAMA* 323 1061–1069. 32031570 10.1001/jama.2020.1585 PMC7042881

[B110] Wang X. Ye Y. Gong H. Wu J. Yuan J. Wang S. (2016). The effects of different angiotensin II type 1 receptor blockers on the regulation of the ACE-AngII-AT1 and ACE2-Ang(1-7)-Mas axes in pressure overload-induced cardiac remodeling in male mice. *J. Mol. Cell Cardiol.* 97 180–190. 27210827 10.1016/j.yjmcc.2016.05.012

[B111] Watson T. Goon P. K. Lip G. Y. (2008). Endothelial progenitor cells, endothelial dysfunction, inflammation, and oxidative stress in hypertension. *Antioxid. Redox. Signal.* 10 1079–1088. 18315493 10.1089/ars.2007.1998

[B112] Wu C. Chen X. Cai Y. Xia J. Zhou X. Xu S. (2020). Risk factors associated with acute respiratory distress syndrome and death in patients with coronavirus disease 2019 pneumonia in Wuhan, China. *JAMA Intern Med.* 180 934–943. 32167524 10.1001/jamainternmed.2020.0994 PMC7070509

[B113] Yang G. Tan Z. Zhou L. Yang M. Peng L. Liu J. (2020). Effects of angiotensin II receptor blockers and ACE (Angiotensin-Converting Enzyme) inhibitors on virus infection, inflammatory status, and clinical outcomes in patients with COVID-19 and hypertension: a single-center retrospective study. *Hypertension* 76 51–58. 32348166 10.1161/HYPERTENSIONAHA.120.15143

[B114] Zanoli L. Briet M. Empana J. P. Cunha P. G. Mäki-Petäjä K. M. Protogerou A. D. (2020). Vascular consequences of inflammation: a position statement from the ESH Working Group on Vascular Structure and Function and the ARTERY Society. *J. Hypertens.* 38 1682–1698. 32649623 10.1097/HJH.0000000000002508 PMC7610698

[B115] Zhang J. Chen Q. Zhong J. Liu C. Zheng B. Gong Q. (2019). DPP-4 inhibitors as potential candidates for antihypertensive therapy: improving vascular inflammation and assisting the action of traditional antihypertensive drugs. *Front. Immunol.* 10:1050. 10.3389/fimmu.2019.01050 31134095 PMC6526751

[B116] Zhang J. J. Dong X. Cao Y. Y. Yuan Y. D. Yang Y. B. Yan Y. Q. (2020). Clinical characteristics of 140 patients infected with SARS-CoV-2 in Wuhan, China. *Allergy* 75 1730–1741. 32077115 10.1111/all.14238

[B117] Zhang L. H. Pang X. F. Bai F. Wang N. P. Shah A. I. Mckallip R. J. (2015). Preservation of glucagon-like peptide-1 level attenuates angiotensin II-induced tissue fibrosis by altering AT1/AT 2 receptor expression and angiotensin-converting enzyme 2 activity in rat heart. *Cardiovasc. Drugs. Ther.* 29 243–255. 25994830 10.1007/s10557-015-6592-7

[B118] Zhong J. Rao X. Deiuliis J. Braunstein Z. Narula V. Hazey J. (2013). A potential role for dendritic cell/macrophage-expressing DPP4 in obesity-induced visceral inflammation. *Diabetes Metab. Res. Rev.* 62 149–157. 10.2337/db12-0230 PMC3526020 22936179

[B119] Zhou F. Yu T. Du R. Fan G. Liu Y. Liu Z. (2020a). Clinical course and risk factors for mortality of adult inpatients with COVID-19 in Wuhan, China: a retrospective cohort study. *Lancet* 395 1054–1062. 32171076 10.1016/S0140-6736(20)30566-3 PMC7270627

[B120] Zhou P. Yang X. L. Wang X. G. Hu B. Zhang L. Zhang W. (2020b). A pneumonia outbreak associated with a new coronavirus of probable bat origin. *Nature* 579 270–273. 32015507 10.1038/s41586-020-2012-7 PMC7095418

[B121] Zhu N. Zhang D. Wang W. Li X. Yang B. Song J. (2020). A novel coronavirus from patients with Pneumonia in China, 2019. *N. Engl. J. Med.* 382 727–733. 31978945 10.1056/NEJMoa2001017 PMC7092803

[B122] Zuin M. Rigatelli G. Zuliani G. Rigatelli A. Mazza A. Roncon L. (2020). Arterial hypertension and risk of death in patients with COVID-19 infection: systematic review and meta-analysis. *J. Infect.* 81 e84–e86. 32283158 10.1016/j.jinf.2020.03.059 PMC7151373

