# Supplementary material for: Biological Context Linking Hypertension and Higher Risk for COVID-19 Severity
Source: Front Physiol. 2020 Nov 19;11:599729. doi: 10.3389/fphys.2020.599729 (PMC7710931; doi:10.3389/fphys.2020.599729)
Supplement: Supplementary file 1 [file Table_1.DOCX]

Supplementary Material

**Biological context linking hypertension and higher risk for COVID-19 severity**

Caio A. M. Tavares^1^, Matthew A. Bailey^2^, Adriana C. C. Girardi^3*^

^1^ Cardiogeriatric Unit, Heart Institute (InCor) – University of São Paulo Medical School, São Paulo, Brazil

^2^Centre for Cardiovascular Science, Queen's Medical Research Institute, The University of Edinburgh, Edinburgh, United Kingdom

^3^Laboratory of Genetics and Molecular Cardiology, Heart Institute (InCor) – University of São Paulo Medical School, São Paulo, SP, Brazil

**SUPPLEMENTARY TABLE**

**Table S1 - Observational studies comparing ACEi/ARBs in patients with COVID-19**

| Reference | Country | Sample size | Comparison group | Outcome | Results (size effect and 95% CI or outcome frequency) | Observations |
| --- | --- | --- | --- | --- | --- | --- |
| (Meng et al., 2020) | China | 42 patients with hypertension | Hypertensive patients on the use of other  medications | Disease severity* | **Severe disease:**  **RAS inhibitors** 23.5%  **Non-RAS inhibitors** 48% | Not adjusted for confounding |
| (Gao et al., 2020) | China | 710 patients with hypertension | Hypertensive patients on the use of other  medications | Mortality | **HR 0.85** (0.28-2.58)  (RAS inhibitors users versus non-users) | Low mortality in both groups (2.2% RAS inhibitors users and 3.6% non-users) |
| (Li et al., 2020) | China | 362 | ACEi/ARBs non-users | Severe disease  Mortality | **Severe disease:**  32.9% vs 30.7% (p=.645)  **Mortality:**  27.3% vs 33.0%; (p = .34)  (RAS inhibitors users versus non-users) | Comparison not between antihypertensive medications |
| (Zhang et al., 2020) | China | 1128 patients with hypertension | ACEi/ARBs non-users | Mortality | **OR 0.37** (0.15-0.89)  (RAS inhibitors users versus non-users)  **OR 0.30** (0.12-0.70)  (RAS inhibitors users versus other antihypertensives) | Propensity score-matched analysis  Medication use during hospitalization |
| (Feng et al., 2020) | China | 476  113 patients with hypertension | 4 groups:  ACEi  ARB,  ACEi or ARBs  Other regimens | Disease severity* | **Severe:**  **ACEi** 12.5%  **ARB** 7.4%  **ACEi/ARBs** 6.1%  **Other regimens** 19.4%  **Critical:**  **ACEi** 0%  **ARB** 7.4%  **ACEi or ARBs** 6.1%  **Other regimens** 24.3% | Only 113 HTN patients:  ACEi 8  ARB 27  ACEi or ARBs 33  Other regimens 62 |
| (Guo et al., 2020) | China | 187 | ACEi/ARBs non-users | Troponin elevation  Mortality | **Troponin elevation:**  ACEi/ARBs:21.1%  Non-users: 5.9%  **Mortality:**  ACEi/ARBs: 38.8%  Non-users: 21.4% | Only 61 patients with HTN and 19 on ACEi/ARB |
| (Yang et al., 2020) | China | 126 patients with hypertension | ACEi/ARBs non-users | Disease severity* | **Severe:**  25.6% vs 19.3%  **Critical:**  9.3 vs. 22.9%  (RAS inhibitors users versus non-users) | 83 patients on ACEi/ARB |
| (Pan et al., 2020) | China | 282 patients with hypertension | ACEi/ARBs non-users | Mortality | 9.8 vs. 26.1%  (RAS inhibitors users versus non-users) | Only 41 patients on ACEi/ARB  Not adjusted for confounding |
| (de Abajo et al., 2020) | Spain | 1139 cases  11390 population controls | Randomly selected 10 individuals per case and matched by sex, age, region and date | COVID-19 requiring hospital admission | **OR 0.94** (0.77-1.15)  (RAS inhibitors users versus users of other antihypertensive drugs) | In-hospital treatment information not available |
| (Reynolds et al., 2020) | USA | 12,594 all patients  4357 patients with hypertension | Propensity-score matching for each medication class | Severe disease or mortality (combined) | **Patients with Hypertension**:24.7% vs 25.3%  **All matched patients:** 24.8% vs 24.9% (RAS inhibitors users versus non-users) | Analysis based on propensity-score models  Ruled out difference ≥10% by RAS inhibitors |
| (Lala et al., 2020) | USA | 2,736 | ACEi/ARBs non-users | Mortality | **OR 1.05** (0.85-1.31)  (RAS inhibitors users versus non-users) | Comparison not between antihypertensive medications |
| (Richardson et al., 2020) | USA | 5,700 | ACEi/ARBs non-users | Mortality | **ACEi**: 32.7%  **ARB**: 30.6%  **No ACEi/ARB**: 26.7% | Outcomes assessed only for 2634 patients (1366 with HTN and 953 on ACEi/ARB) |
| (Gupta et al., 2020) | USA | 2215 ICU patients | ACEi/ARBs non-users | Mortality at 28 days | **ACEi:** 38.4% vs 34.7%  **ARB:** 43% vs. 33.9%  (users vs. non-users) | 1322 patients with Hypertension  ACEi 401  ARB 365  Not adjusted for confounding |
| (Fosbol et al., 2020) | Denmark | 4480 | ACEi/ARBs non-users | Mortality  Disease Severity | **Mortality:** HR 0.83 (0.67-1.03)  **Severe disease:** HR 1.15 (0.94-1.41)  (RAS inhibitors users versus non-users for both) | Cases defined based on ICD-10 codes |
| (Mancia et al., 2020) | Italy | 6272 case-patients  30,759 controls | Controls randomly selected and matched by sex, age, and residence | Severe disease or mortality (combined) | **OR 0.83** (0.63-1.15) for ARBs and **0.91** (0.69-1.21) for ACEi (versus users of other antihypertensive drugs) | Restricted to individuals > 40 years old |
| (Grasselli et al., 2020) | Italy | 3988 ICU patients | ACEi/ARBs non-users (in multivariable analysis) | Time to death in days | **Multivariable HR**  **ACEi:** 1.17 (0.97-1.42)  **ARB:** 1.05 (0.85-1.29) | 1643 patients with HTN |
| (de Abajo et al., 2020) | Spain | 1139 cases  11390 population controls | Randomly selected 10 individuals per case and matched by sex, age, region and date | COVID-19 requiring hospital admission | **OR 0.94** (0.77-1.15)  (RAS inhibitors users versus users of other antihypertensive drugs) | In-hospital treatment information not available |

*** =**according to National Health Commission of the People's Republic of China guidelines

**SUPPLEMENTARY REFERENCES**

De Abajo, F.J., Rodriguez-Martin, S., Lerma, V., Mejia-Abril, G., Aguilar, M., Garcia-Luque, A., Laredo, L., Laosa, O., Centeno-Soto, G.A., Angeles Galvez, M., Puerro, M., Gonzalez-Rojano, E., Pedraza, L., De Pablo, I., Abad-Santos, F., Rodriguez-Manas, L., Gil, M., Tobias, A., Rodriguez-Miguel, A., Rodriguez-Puyol, D., and Group, M.-a.C.S. (2020). Use of renin-angiotensin-aldosterone system inhibitors and risk of COVID-19 requiring admission to hospital: a case-population study. *Lancet* 395**,** 1705-1714.

Feng, Y., Ling, Y., Bai, T., Xie, Y., Huang, J., Li, J., Xiong, W., Yang, D., Chen, R., Lu, F., Lu, Y., Liu, X., Chen, Y., Li, X., Li, Y., Summah, H.D., Lin, H., Yan, J., Zhou, M., Lu, H., and Qu, J. (2020). COVID-19 with Different Severities: A Multicenter Study of Clinical Features. *Am J Respir Crit Care Med* 201**,** 1380-1388.

Fosbol, E.L., Butt, J.H., Ostergaard, L., Andersson, C., Selmer, C., Kragholm, K., Schou, M., Phelps, M., Gislason, G.H., Gerds, T.A., Torp-Pedersen, C., and Kober, L. (2020). Association of Angiotensin-Converting Enzyme Inhibitor or Angiotensin Receptor Blocker Use With COVID-19 Diagnosis and Mortality. *JAMA*.

Gao, C., Cai, Y., Zhang, K., Zhou, L., Zhang, Y., Zhang, X., Li, Q., Li, W., Yang, S., Zhao, X., Zhao, Y., Wang, H., Liu, Y., Yin, Z., Zhang, R., Wang, R., Yang, M., Hui, C., Wijns, W., Mcevoy, J.W., Soliman, O., Onuma, Y., Serruys, P.W., Tao, L., and Li, F. (2020). Association of hypertension and antihypertensive treatment with COVID-19 mortality: a retrospective observational study. *Eur Heart J* 41**,** 2058-2066.

Grasselli, G., Greco, M., Zanella, A., Albano, G., Antonelli, M., Bellani, G., Bonanomi, E., Cabrini, L., Carlesso, E., Castelli, G., Cattaneo, S., Cereda, D., Colombo, S., Coluccello, A., Crescini, G., Forastieri Molinari, A., Foti, G., Fumagalli, R., Iotti, G.A., Langer, T., Latronico, N., Lorini, F.L., Mojoli, F., Natalini, G., Pessina, C.M., Ranieri, V.M., Rech, R., Scudeller, L., Rosano, A., Storti, E., Thompson, B.T., Tirani, M., Villani, P.G., Pesenti, A., Cecconi, M., and Network, C.-L.I. (2020). Risk Factors Associated With Mortality Among Patients With COVID-19 in Intensive Care Units in Lombardy, Italy. *JAMA Intern Med*.

Guo, T., Fan, Y., Chen, M., Wu, X., Zhang, L., He, T., Wang, H., Wan, J., Wang, X., and Lu, Z. (2020). Cardiovascular Implications of Fatal Outcomes of Patients With Coronavirus Disease 2019 (COVID-19). *JAMA Cardiol*.

Gupta, S., Hayek, S.S., Wang, W., Chan, L., Mathews, K.S., Melamed, M.L., Brenner, S.K., Leonberg-Yoo, A., Schenck, E.J., Radbel, J., Reiser, J., Bansal, A., Srivastava, A., Zhou, Y., Sutherland, A., Green, A., Shehata, A.M., Goyal, N., Vijayan, A., Velez, J.C.Q., Shaefi, S., Parikh, C.R., Arunthamakun, J., Athavale, A.M., Friedman, A.N., Short, S.a.P., Kibbelaar, Z.A., Abu Omar, S., Admon, A.J., Donnelly, J.P., Gershengorn, H.B., Hernan, M.A., Semler, M.W., Leaf, D.E., and Investigators, S.-C. (2020). Factors Associated With Death in Critically Ill Patients With Coronavirus Disease 2019 in the US. *JAMA Intern Med*.

Lala, A., Johnson, K.W., Januzzi, J.L., Russak, A.J., Paranjpe, I., Richter, F., Zhao, S., Somani, S., Van Vleck, T., Vaid, A., Chaudhry, F., De Freitas, J.K., Fayad, Z.A., Pinney, S.P., Levin, M., Charney, A., Bagiella, E., Narula, J., Glicksberg, B.S., Nadkarni, G., Mancini, D.M., Fuster, V., and Mount Sinai Covid Informatics, C. (2020). Prevalence and Impact of Myocardial Injury in Patients Hospitalized with COVID-19 Infection. *J Am Coll Cardiol*.

Li, J., Wang, X., Chen, J., Zhang, H., and Deng, A. (2020). Association of Renin-Angiotensin System Inhibitors With Severity or Risk of Death in Patients With Hypertension Hospitalized for Coronavirus Disease 2019 (COVID-19) Infection in Wuhan, China. *JAMA Cardiol*.

Mancia, G., Rea, F., Ludergnani, M., Apolone, G., and Corrao, G. (2020). Renin-Angiotensin-Aldosterone System Blockers and the Risk of Covid-19. *N Engl J Med*.

Meng, J., Xiao, G., Zhang, J., He, X., Ou, M., Bi, J., Yang, R., Di, W., Wang, Z., Li, Z., Gao, H., Liu, L., and Zhang, G. (2020). Renin-angiotensin system inhibitors improve the clinical outcomes of COVID-19 patients with hypertension. *Emerg Microbes Infect* 9**,** 757-760.

Pan, W., Zhang, J., Wang, M., Ye, J., Xu, Y., Shen, B., He, H., Wang, Z., Ye, D., Zhao, M., Luo, Z., Liu, M., Zhang, P., Gu, J., Liu, M., Li, D., Liu, J., and Wan, J. (2020). Clinical Features of COVID-19 in Patients With Essential Hypertension and the Impacts of Renin-angiotensin-aldosterone System Inhibitors on the Prognosis of COVID-19 Patients. *Hypertension* 76**,** 732-741.

Reynolds, H.R., Adhikari, S., Pulgarin, C., Troxel, A.B., Iturrate, E., Johnson, S.B., Hausvater, A., Newman, J.D., Berger, J.S., Bangalore, S., Katz, S.D., Fishman, G.I., Kunichoff, D., Chen, Y., Ogedegbe, G., and Hochman, J.S. (2020). Renin-Angiotensin-Aldosterone System Inhibitors and Risk of Covid-19. *N Engl J Med* 382**,** 2441-2448.

Richardson, S., Hirsch, J.S., Narasimhan, M., Crawford, J.M., Mcginn, T., Davidson, K.W., And the Northwell, C.-R.C., Barnaby, D.P., Becker, L.B., Chelico, J.D., Cohen, S.L., Cookingham, J., Coppa, K., Diefenbach, M.A., Dominello, A.J., Duer-Hefele, J., Falzon, L., Gitlin, J., Hajizadeh, N., Harvin, T.G., Hirschwerk, D.A., Kim, E.J., Kozel, Z.M., Marrast, L.M., Mogavero, J.N., Osorio, G.A., Qiu, M., and Zanos, T.P. (2020). Presenting Characteristics, Comorbidities, and Outcomes Among 5700 Patients Hospitalized With COVID-19 in the New York City Area. *JAMA*.

Yang, G., Tan, Z., Zhou, L., Yang, M., Peng, L., Liu, J., Cai, J., Yang, R., Han, J., Huang, Y., and He, S. (2020). Effects Of ARBs And ACEIs On Virus Infection, Inflammatory Status And Clinical Outcomes In COVID-19 Patients With Hypertension: A Single Center Retrospective Study. *Hypertension*.

Zhang, P., Zhu, L., Cai, J., Lei, F., Qin, J.J., Xie, J., Liu, Y.M., Zhao, Y.C., Huang, X., Lin, L., Xia, M., Chen, M.M., Cheng, X., Zhang, X., Guo, D., Peng, Y., Ji, Y.X., Chen, J., She, Z.G., Wang, Y., Xu, Q., Tan, R., Wang, H., Lin, J., Luo, P., Fu, S., Cai, H., Ye, P., Xiao, B., Mao, W., Liu, L., Yan, Y., Liu, M., Chen, M., Zhang, X.J., Wang, X., Touyz, R.M., Xia, J., Zhang, B.H., Huang, X., Yuan, Y., Rohit, L., Liu, P.P., and Li, H. (2020). Association of Inpatient Use of Angiotensin Converting Enzyme Inhibitors and Angiotensin II Receptor Blockers with Mortality Among Patients With Hypertension Hospitalized With COVID-19. *Circ Res*.
